# Supporting LGBTQ+ Students: A Focus Group Study with Junior High School Nurses

**DOI:** 10.1177/10598405221086035

**Published:** 2022-03-18

**Authors:** Minna Laiti, Anni Pakarinen, Heidi Parisod, Mark Hayter, Salla Sariola, Sanna Salanterä

**Affiliations:** 1 Department of Nursing Science, 60654 University of Turku, Turku, Finland; 2 281649 The Nursing Research Foundation, Helsinki, Finland; 3 Faculty of Health, Psychology & Social Care, 5289 Manchester Metropolitan University, Manchester, UK; 4 Faculty of Political Sciences, Discipline of Sociology, 3835 University of Helsinki, Helsinki, Finland; 5 60652 Turku University Hospital, Turku, Finland

**Keywords:** school nurse knowledge/perceptions/self-efficacy, middle/junior/high school, qualitative research, focus groups, sexual minority youth, gender minority youth, family life/sexuality

## Abstract

LBGTQ+ students often miss the support and information they need in the school nursing, but little is known about junior high school (JHS) nurses’ work with LGBTQ+ students. 15 JHS nurses were interviewed in focus groups about their perceptions of supporting LGBTQ+ students. Four interconnected themes were identified with inductive thematic analysis: (1) JHS nurses’ professional identity and practice; (2) Recognition of sexual and gender diversity in school; (3) Family acceptance process; and (4) LGBTQ+ students as school nursing clients. JHS nurses self-identified as accepting professionals, but having limited skills, knowledge, and education needed in supporting LGBTQ+ students. Supporting LGBTQ+ students is a complex phenomenon, and to enhance JHS nurses’ competence in providing care for these students, sexual and gender diversity needs to be included in evidence-based nursing information sources, covered in nursing education, and the school needs to be secured as LGBTQ+ safe place.

## Background

Gender identity formations take place in adolescence (Bussey, 2011). However, transgender, and other gender minority adolescents are often less visible, in a more unequal position compared to cisgender peers (*a person whose gender identity conforms with sex assigned at birth*) in health services (Mullinax et al., 2017), and have unmet health care needs (Laiti et al., 2019; Rafferty et al., 2018). Sexual health is closely linked to adolescents’ human rights regardless of their gender or sexual orientation (Mullinax et al., 2017; World Health Organization, 2006). Also, sexual minority adolescents have experiences of getting inadequate sexual health services from health professionals (Laiti et al., 2019; Rose & Friedman, 2013). These aspects may challenge school nurses, who are expected to provide equally preventive, curative, supportive care, and health promotion to school-aged children and adolescents (WHO, 2021; WHO Regional Office for Europe, 2014).

In Finland, school nursing is a free school-based health service organized by municipalities (Government Degree, 338/2011). One junior high school (JHS) nurse is usually responsible for about 600 students’ health care (Wiss et al., 2018), between 13–15 years old. Their work regularly involves health checks and discussions about physical changes, sexual health, sexuality, and relationships with students. (Finnish Institute for Health & Welfare, 2019; Finnish National Agency for Education, 2014).

Studies concerning sexual and gender diversity in school nursing are scarce. Research on LGBTQ+ (*lesbian, gay, bisexual, trans, queer or questioning, and other*) students’ perspective indicates school nurses’ attitudes vary from supportive to unsupportive, school nurses’ knowledge about sexual and gender diversity can be limited, and school nurses lack consistent information about sexual and gender diversity (Laiti et al., 2021; Rasberry et al., 2015; Rose & Friedman, 2017). Research on school professionals’ preparedness to support LGBTQ+ students is minimal (Mahdi et al., 2014; Sawyer et al., 2006). Research focusing on school nurses’ experiences and perceptions about the topic is crucial, as school nurses can be advocates for LGBTQ+ students and support their health and growth. The promotion of safe and inclusive school environments can have substantial effects on discrimination due to sexual orientation or gender identity, which causes LGBTQ+ students’ higher rates of mental health issues and suicidal behavior (Council of Europe, 2018; European Union Agency for Fundamental Rights, 2020).

Nursing students have reported inadequate education and training in LGBTQ+ patient care knowledge (Richardson et al., 2017), and graduated nurses can feel uncomfortable with LGBTQ+ health issues due to this lack of knowledge and training (Carabez et al., 2015; Rider et al., 2019). Since this phenomenon is under-researched in school nursing, it is important to explore, how JHS nurses perceive working and supporting LGBTQ+ students. The purpose of our study was to describe JHS nurses’ perceptions of supporting LGBTQ+ students.

## Methods

We conducted a qualitative descriptive study to explore the phenomenon. The study was done in four Southern Finland municipalities. The Ethics Committee of the University of Turku approved the study (6/2019).

### Sampling and Data Collection

We used purposeful sampling to reach JHS nurses, who can give in-depth insights about the topic (Patton, 2015a). The eligibility criteria were: (1) working experience with JHS-aged students, and (2) mother tongue was Finnish or Swedish. JHS nurses meeting the eligibility criteria and willing to participate received written information about the study and completed a consent form.

A semi-structured interview guide (Table 1) was developed based on our study on LGBTQ+ students’ experiences of JHS nursing (Laiti et al., 2021). In that study, LGBTQ+ students described sexual and gender diversity in the contexts of JHS nurse's personal and professional characteristics, but also junior high school, peer attitudes, family issues, and societal attitudes towards LGBTQ+ people. These topics formed the main questions in the guide, and two fictional warmup stories were added to stimulate participants’ discussion to the topic (Patton, 2015b). We collected additional data on educational background and working experience/context of JHS nurses. Four focus groups were undertaken in 2019. Three focus groups consisted of three JHS nurses and one consisted of six JHS nurses (N = 15). Focus group lasted from 49 to 65 min. Interviews were audio-recorded, transcribed verbatim and transcriptions were uploaded to NVivo® Version 12. Two authors (ML, SR) cross-checked the data for accurate translations between Finnish and English.

**Table 1.** Focus Group Interview Guide.

**Warmup stories about cases of engagement with LGBTQ+ students in JHS nursing**
1. A student, who resembles a girl to you comes to your office. The student starts to tell you that she has started to consider her gender identity. The student feels that they are not identifying as their sex assigned at birth (girl). The student is visibly anxious about how her body is becoming more feminine due to puberty, and that her body doesn't correspond with their gender identity. *How would you act/discuss with the student?*
2. A student comes to your office, wanting to discuss sexual health issues. It turns out that the student dates a same-sex partner (in other words, the student's sexual orientation might be lesbian, gay, or bisexual). The student seems a bit shy and nervous after disclosing their identity to you, but express they don't know how to practice safe sex with their partner. *How would you discuss about the topic with the student?*
**Questions to be discussed after warmup stories**
1. What are contemporary adolescents like, in your opinion, with respect sexuality and gender?
2. How is sexual and gender diversity in adolescence reflected in your work when engaging with students? Have you, for example, met students whose sexual orientation is other than heterosexual, or adolescents whose gender identity is other than girl/boy? Are these topics discussed in your further education, or in other contexts?
3. What kinds of thoughts do social discourses about sexual and gender diversity evoke in you as a JHS nurse?
4. Does junior high school as an organization support discussion about sexual and gender diversity? If you think *yes*, could you tell me how, and if *no*, could you tell your perceptions about why the situation is like that?
5. Does your supervisor support the discussion about sexual and gender diversity? If *yes*, could you tell me how, if *no*, could you tell your perceptions about why the situation is like that?
6. How do you perceive working with parents regarding sexual and gender diversity? For example, in a situation where the student has disclosed their identity only to you, and not to their parents?
7. Is there something you would like to add to the discussions we have had?

### Data Analysis

We used inductive thematic analysis described by Braun and Clarke (2006). After uploading data to NVivo®, the corresponding author (ML) familiarized the data by reading it through several times, coded the data, and generated ideas about the themes. Initial themes were created and presented as an initial thematic map with descriptions. Three authors (ML, AP, HP) reviewed the initial map by comparing themes, codes, and descriptions together. Some codes were regrouped into other themes, some themes were collapsed into others, and new themes were created. Example of data analysis process is presented in Table 2.

**Table 2.** Thematic Data Analysis Process.

Data analysis process and theme generation			
Original data	Code(s) generated from data	Subthemes	Main theme
*JHS nurse 1: Well I think it is discussion and that school nursing is a neutral place where you can, if the student wants, discuss about anything without that family gets to know about your private matters*. *JHS nurse 3: And acceptance that everyone can be just who they are […]* *JHS nurse 1: Indeed, that there's no right or wrong, or that you should be something, and then we can together start think about what would support the student, and how to proceed.*	Empathetic school nurse Diversity accepting school nurse Respecting student's rights Confidentiality Open and accepting discussion Identifying student's needs	Attitudes and values towards LGBTQ + LGBTQ+ supportive nursing activities	**JHS nurse's professional identity and practice**

## Results

**Figure 1.**
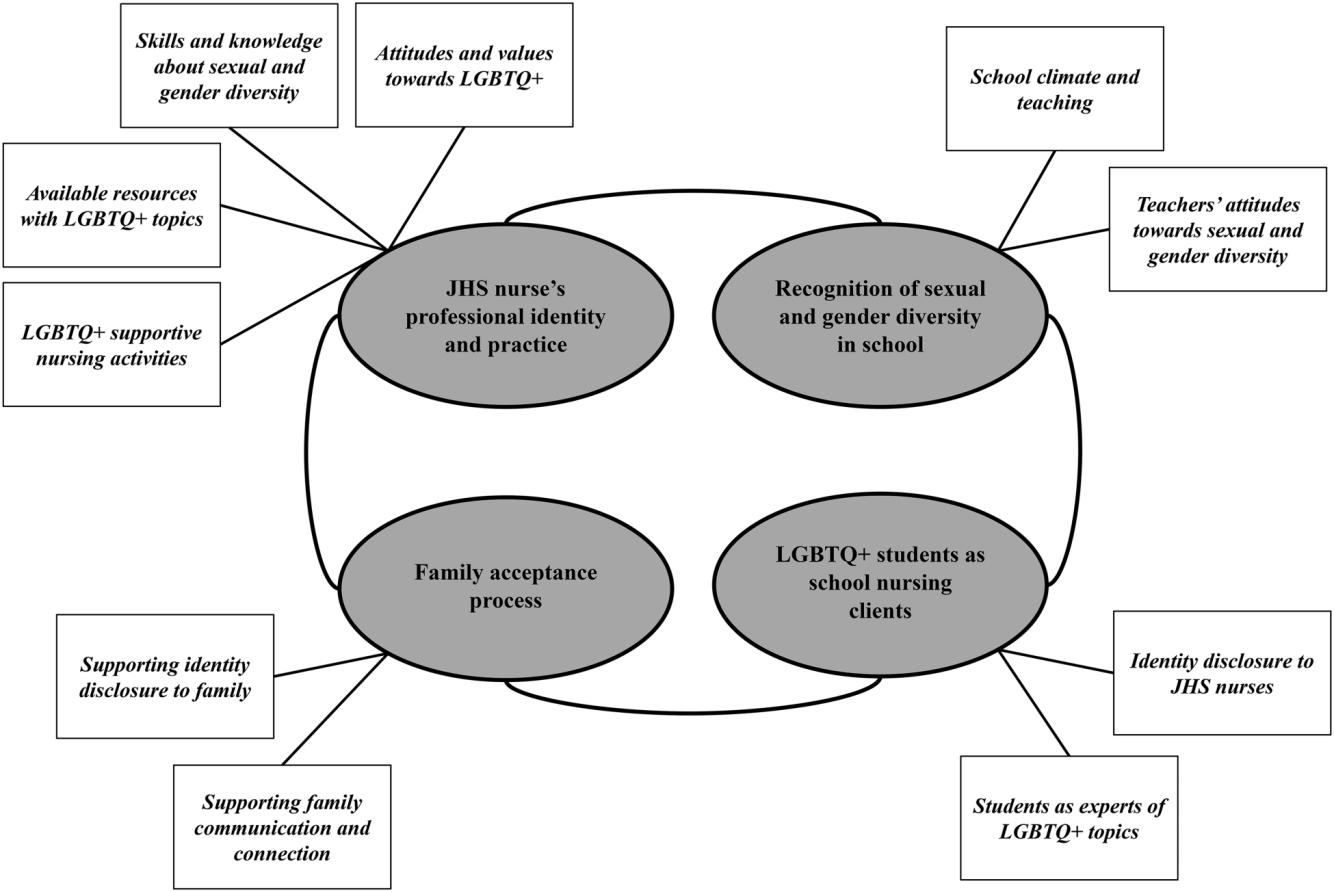
Supporting LGBTQ+ students in JHS nursing.

Half of JHS nurses had a bachelor's degree in public health nursing (n = 8), and other half (n = 7) had a vocational qualification of public health nurse. The working experience with JHS-aged students ranged from six months to 29 years. The number of schools the JHS nurses oversaw varied between one and three. The JHS nurses described four main themes regarding supporting LGBTQ+ students in the JHS nursing: *JHS nurse's professional identity and practice; Recognition of sexual and gender diversity in school*; *Family acceptance process; LGBTQ+ students as school nursing clients* (see Figure 1)*.* Each main theme included subthemes, which we present in the following paragraphs.

### JHS Nurse's Professional Identity and Practice

When supporting LGBTQ+ students in their work, JHS nurses discussed their professional identity and practice from various points. This theme included four subthemes: *Attitudes and values towards LGBTQ+*; *Skills and knowledge about sexual and gender diversity*; *Available resources with LGBTQ+ topics; LGBTQ+ supportive nursing activities***.**

#### Attitudes and Values Towards LGBTQ+

JHS nurses showed acceptance and empathy towards LGBTQ+ students. Acceptance and empathy were described as expressing and discussing sexual and gender diversity open-mindedly. When discussing attitudes towards LGBTQ+ students, one of JHS nurses said:

JHS nurse 3: *And acceptance so that everyone can be just the way they are. […] I think that's like totally normal [sexual and gender diversity]. Anybody can like anyone [romantically or sexually].* (Focus group 3)

JHS nurses stated that open-mindedness was a prerequisite for creating a safe space for LGBTQ+ students, and they conveyed it actively. Without open-mindedness, students might not open to JHS nurses, or consider JHS nurse as trustworthy.

Professional values guided JHS nurses’ work. JHS nurses respected LGBTQ+ students’ rights and highlighted the importance of confidentiality. Students’ rights to privacy and autonomy were essential, which also applied to working with students’ parents.

JHS nurse 4: *We have quite many students and situations, where we need to agree [with the student] if certain topics can be discussed while the parent is present*.

JHS nurse 2: *And I consider it positive, when the student has at least some way been brave enough to tell me about it. […] I think it's better than the student would be alone with that issue, and it would worry them.* (Focus group 1)

JHS nurses highlighted, that it was never acceptable to neglect students’ rights, especially with sexual orientation and gender identity, which can be intimate and sensitive topics to students.

#### Skills and Knowledge About Sexual and Gender Diversity

JHS nurses reflected constructively on their skills and knowledge, and commented it was okay to admit their knowledge gaps to LGBTQ+ students. In such instances, they searched for additional information about the topic.

JHS nurse 3: *[…] you can always tell if you don't know something […] I would ask the student to come again to my office, and before that I would learn more about where they could get support to their sexuality, and to things they are wondering […]* (Focus group 2)

It was crucial to meet the student again to share the information JHS nurses have found. When discussing knowledge gaps, JHS nurses were sometimes unfamiliar with the STI prevention for female couples since they were more attuned to discuss about condoms and pills. Furthermore, they had only condoms to distribute. However, JHS nurses highlighted they were willing to educate themselves on sexual and gender diversity topics.

#### Available Resources with LGBTQ+ Topics

JHS nurses mentioned several professionals who they can consult for LGBTQ+ topics. In schools, they could consult a social worker, psychiatric nurse, or sex educator. Outside schools, JHS nurses consulted health professionals experienced with LGBTQ+ topics, especially when they had limited skills, resources, or time to support LGBTQ+ students.

JHS nurse 3: *Here, we have a local youth clinic, where we may refer the student, if they have anxiety, maybe depression, maybe some other mood related symptoms […] In that youth clinic, there are [professionals] educated on gender identity topics […]* (Focus group 4)

JHS nurses knew information sources for sexual and gender diversity, however, most of them were organisation-based.

JHS nurse 3: *And there aren't many resources, which are specialized to these topics […] Probably they are mostly [LGBTQ+ human rights] organisations but not ‘real ones’, like, evidence-based information sources about the topic […].* (Focus group 1)

Evidence-based information sources were crucial to JHS nurses’ work, and nurses especially missed those targeted toward nursing professionals. Information sources for same-sex couples protection, especially for girls, were also unfamiliar to some JHS nurses.

JHS nurses’ supervisors in the social and health care department and nursing manager were willing to support their participation in further education. However, most of the education needed to be free-of-charge due to limited funds.

Researcher: *Is all further education subject to a charge?*

JHS nurse 4: *Noo! For example, university hospital organises education, but there isn't much about this topic, there's more education about child development, diabetes care, and so on.* (Focus group 1)

Professional and free-of-charge education about sexual and gender diversity was rare. Nevertheless, when available, JHS nurses gained substantial benefit from the education on sexuality and gender, LGBTQ+ terminology, and how to sensitively discuss sexuality and gender identity. They shared that sexual and gender diversity was only briefly taught in the nursing education, or not at all. Nursing education was perceived as developing at slower pace than societal understanding of LGBTQ+ populations. Including sexual and gender diversity in education was seen as essential for all health professionals.

#### LGBTQ+ Supportive Nursing Activities

JHS nurses mentioned several nursing activities when supporting LGBTQ+ students: identifying student's needs; providing sexual health and relationship counseling; and supporting mental health. Identifying students’ needs occurred when interviewing students and giving space and time to students to describe their situation.

JHS nurse 1: *I suppose [I would ask] when she has noticed [gender dysphoria], what kinds of things and why is she experiencing so, how is she experiencing deviating from gender norms and […] has she had possibilities to discuss with someone, and does she have some expectations [about the future].* (Focus group 3)

The identification of needs helped JHS nurses to decide, whether the student needed information, a trustful conversation partner, an advocate, or LGBTQ+ specialized health services.

Besides identifying students’ needs, sexual health and relationship counseling was an essential JHS nursing activity. Counseling was done with an assumption-free approach and systematically with all students.

JHS nurse 1: *And it is important […] to discuss, not only protection in hetero sex, but all different STIs in a general level, it doesn't mean that it must be woman and man, or girl and boy, but it [protection] needs to be taken care of […]* (Focus group 4)

Sexuality was understood as a multidimensional part of health and wellbeing, which develops individually throughout life. JHS nurses considered diverse sexual orientations as normal in sexuality.

Furthermore, mental health was a common topic in JHS nursing, and JHS nurses identified having good skills for supporting LGBTQ+ students’ mental health. Supporting mental health was significant especially for students experiencing gender dysphoria.

JHS nurse 1: *[…] when you feel different than how you’ve been raised, and for example, what surrounding world assumes you to be […] and for example probably causes you depressive symptoms, panic, and anxiety […] [JHS nurse wants to find out] how to support self-esteem and kind of positivity in life […]* (Focus group 2)

JHS nurses could understand how a minority status can affect the mental health of LGBTQ+ students and nurses treated students with empathy. If they had limited time to support the student, they referred them to a psychiatric nurse.

### Recognition of Sexual and Gender Diversity in School

The recognition of sexual and gender diversity in school was related to the work of JHS nurses, since JHS nurses’ practice was in schools, and JHS nurses were school personnel. This main theme included two subthemes: *School climate and teaching*; and *Teachers’ attitudes towards sexual and gender diversity*.

#### School Climate and Teaching

JHS nurses described differences among schools’ climate; some schools were LGBTQ+ inclusive, while others were still hetero- and gender-normative. In LGBTQ+ inclusive schools, gender minority students’ needs were respected (e.g. gender neutral bathrooms), and equality and diversity were discussed with students. In hetero- and gender-normative schools, students were divided into girl/boy groups, and students were assumed as heterosexual and cis-gendered.

JHS nurse 1: *Well, there are a lot of historical traditions in the school, like, grouping girls together and boys together in PE […]*

JHS nurse 2: *Yeah, and girls have handicraft, and boys have wood craft classes […]* (Focus group 1)

School as an institution was seen conservative. JHS nurses perceived that the Finnish school system is not yet comprehensively inclusive, and LGBTQ+ inclusive school climate was more a special effort in school.

When discussing teaching about sexuality and gender identity in school, JHS nurses described how it was mostly teacher-led in sex education and health education classes. The topics covered in these classes did not systematically include sexual and gender diversity.

JHS nurse 1: *It depends on umm…there can be teachers who skip it [sexual and gender diversity] totally, by referring, for example, to their own beliefs.* (Focus group 1)

JHS nurse 2: *And when I have met 9th graders in health check-ups, they have said STIs and contraception are well covered in the classes […]* (Focus group 4)

JHS nurses perceived that teaching included mostly discussions about heterosexual sexual health, and if something about diversity was included, it was about sexual orientations. Trans-related topics were rarely taught to students.

#### Teachers’ Attitudes Towards Sexual and Gender Diversity

Teachers’ attitudes varied towards diversity. Progressive teachers respected students’ identities, talked about equality, and collaborated with JHS nurses. JHS nurses had also met teachers with conservative and out-dated attitudes. These teachers were judgmental towards LGBTQ+ identities, and they expressed it openly.

JHS nurse 2: *We had a substitute who was lesbian and open about it. And she dressed in a masculine way […] And one of our teachers said to her ”Well you could at least sometimes wear women's clothes”.* (Focus group 3)

JHS nurses stated that conservative attitudes could occur even among younger teachers, and their attitudes influenced whether LGBTQ+ students perceived the school as safe for them.

### Family Acceptance Process

Family acceptance was an essential part of supporting LGBTQ+ students. They understood that LGBTQ+ students can have stress about the family acceptance, and how important it was for students. JHS nurses discussed two subthemes within family acceptance: *Supporting identity disclosure to family*; and *Supporting family communication and connection*.

#### Supporting Identity Disclosure to Family

LGBTQ+ students might be concerned about how their family may react to their identity disclosure, and some students shared experiences of conflicts related to their gender expression.

JHS nurse 2: *She came to open up, related to her appearance, that her mother always preferred her to have long hair, and now she had decided to cut very short hair, like boyish style […] she asked what did I think about it, and I said it's everyone's own business, but she had strict discussion at home about it, and then she started to tell it's actually something more than just her hair.* (Focus group 1)

JHS nurses self-identified as a safe adult in family issues. They showed support by listening to LGBTQ+ students, while respecting student's needs, privacy, and autonomy.

#### Supporting Family Communication and Connection

JHS nurses realized that family members might react negatively to LGBTQ+ student's identity and gender expression and were prepared to discuss sexual and gender diversity with the family.

JHS nurse 3: *Sometimes there's a situation, where a parent has difficulties accepting this [LGBTQ+ student's identity]. Even though the girl came terms with it, and didn't identify as a girl, her mother might comment that her identity would be just a phase […] but the family needs to be in the picture. You cannot leave the girl all alone with it.* (Focus group 3)

Discussion with the family was based on being an advocate to LGBTQ+ student. When engaging the topic with the family, it was essential to emphasize the normality of sexual and gender diversity in human life and the importance of family support to LGBTQ+ students’ wellbeing.

### LGBTQ+ Students as School Nursing Clients

JHS nurses discussed also LGBTQ+ students as school nursing clients, and what special aspects were included to engage them as clients. This theme included two subthemes: *Students as experts of LGBTQ+ topics*; and *Identity disclosure JHS nurses*.

#### Students as Experts of LGBTQ+ Topics

LGBTQ+ students were active information searchers, and they possibly knew more than nurses about sexual and gender diversity. JHS nurses described how knowledge about diversity can influence students’ self-confidence positively, and nurses regarded them as experts of diversity topics and their own life.

JHS nurse 3: *[JHS nurse can ask from the student] can you tell me about it, and they’re very active to tell, and for some students it's the pride to guide us.* (Focus group 4)

JHS nurses acknowledged the information LGBTQ+ students shared with them, and it was a good way to learn more about sexual and gender diversity. In that way, JHS nurses could develop as professionals, and offer more information and support in future engagements with LGBTQ+ students.

#### Identity Disclosure to JHS Nurses

Despite being self-confident, JHS nurses identified that LGBTQ+ students were cautious with the identity disclosure. Cautiousness derived from concerns about nurses’ attitudes to sexual and gender diversity, and LGBTQ+ students first wanted to ensure JHS nurses had accepting attitudes.

JHS nurse 2: *I guess he was seeking for trust and assessing how I would react to different things. Because, you know, they [students] tend to assess what kind of person that is, what topics may shock that person, and what that person perceives as normal […]* (Focus group 3)

JHS nurses considered that attitude testing was part of building a safe and confidential relationship with LGBTQ+ students. The school climate was also recognized for its influence on the extent to which LGBTQ+ students felt safe disclosing their identities to school nurses. This was understandable to JHS nurses, and they were willing to do their best in creating a safe and confidential relationship with students.

## Discussion

Our study explored supporting LGBTQ+ students in school nursing from JHS nurses’ perspective, which has been an under-researched topic. Our findings created an understanding about how JHS nurses self-identified as health professionals, who can offer significant support to LGBTQ+ students. However, alongside their professional identity and practice, the nurses needed to consider the junior high school, students’ family, and students as school nursing clients.

JHS nurses discussed their work from several viewpoints, including their attitudes, values, skills and knowledge, available resources, and nursing activities in supporting LGBTQ+ students. They expressed empathy and acceptance to LGBTQ+ students and understood how creating a safe space was essential in building a confidential relationship with LGBTQ+ students. Previous research supports this perception, since LGBTQ+ youth have identified safe space characteristics (e.g. rainbow flags, leaflets about sexual and gender diversity, open-minded attitudes from professionals) as elements of inclusive health care (Laiti et al., 2019). Furthermore, younger LGBTQ+ generations have become more conscious of their needs and rights in health care, and they expect inclusive practices and engagement when accessing health care services (Newman et al., 2020). This raises up the importance of health services and school nursing to be inclusive of sexual orientation and gender identity.

JHS nurses highlighted an issue of available resources for LGBTQ+ topics. They expressed a clear lack of evidence-based, nursing-focused information sources about sexual and gender diversity. Furthermore, they had limited education options, and nursing education was seen as insufficient in covering sexual and gender diversity. LGBTQ+ students also identified lack of diversity-affirming information, discussion, and protection methods in the JHS nursing (Rasberry et al., 2015; Laiti et al., 2021). A surprising finding in our study was that although JHS nurses considered themselves accepting and open-minded, they expressed a need for training on how to discuss sensitively sexuality and gender identity. The lack of confidence in discussing LGBTQ+ topics has been recognized by nursing students (Richardson et al., 2017). Graduated nurses have also indicated discomfort with gender identity issues (Carabez et al., 2015; Rider et al., 2019). A recent study about providing care for transgender and gender diverse students (Neiman et al., 2023) reported that school nurses lacked the knowledge and resources to share with students and their parents about gender identity topics. Therefore, JHS nurses, other nursing professionals, and nursing students need systematic, evidence-based education about sexual and gender diversity, and engaging LGBTQ+ populations in health care. More available resources of diversity-affirming information are also needed since this is recognized by both LGBTQ+ students (Rasberry et al., 2015; Laiti et al., 2021) and school nurses (Neiman et al., 2023) internationally.

When JHS nurses discussed school as an institution, they stressed there was variation of how LGBTQ+ inclusive schools were, and the inclusion of sexual and gender diversity was rather a special effort than embedded in school climate and practices. Despite including equality and diversity guidelines in Finland's National Core Curriculum (Finnish National Agency for Education, 2014), some schools still maintain hetero- and gender-normative views of sexuality, gender, and to students’ identities. JHS nurses also noted, that teaching did somewhat cover sexual diversity, but not gender diversity or trans-related topics. This insufficiency may be linked to the National Core Curriculum guidelines (Finnish National Agency for Education, 2014) since despite including sexual diversity in sexual health education, there is no clear reference for teaching gender diversity. Hetero- and gender-normative schools have been reported being discriminatory to LGBTQ+ students, and it can affect how safe LGBTQ+ students experience the school environment (Kosciw et al., 2020). Thus, the promotion of safe and inclusive school is not only school nurses’ responsibility, but for all school professionals. Historical traditions, which JHS nurses perceived to be one reason behind the school climate, need to be restructured by following the Finnish National Agency for Education equality guidelines (2014), and ensure children's right to education that supports their personality development (United Nations Convention on the Rights of the Child, 1989).

JHS nurses described having experiences with LGBTQ+ students’ worries about self-expression and identity disclosure to the family. JHS nurses identified this as a risk to student's wellbeing, and they adapted a role of safe adult and advocate in the family acceptance process. It was essential to respect LGBTQ+ students’ rights, and to collaborate with the student and the family. JHS nurses recognized family members may need information about sexual and gender diversity, and the importance of family acceptance to LGBTQ+ students. Through information and discussion, family members could understand their child's sexual orientation or gender identity, and the acceptance process would progress. These results are similar with the study of Neiman et al. (2023) who found that school nurses strongly embrace bridging the gap between transgender and gender diverse students and their families. Furthermore, an accepting child-parent relationship is essential to prevent LGBTQ+ students’ risk for mental health problems (Newcomb et al., 2019). Therefore, including family in discussions about sexual and gender diversity is crucial for supporting LGBTQ+ students’ connection to their families and promoting the family acceptance to their child's identity.

When discussing about LGBTQ+ students as adolescent clients in JHS nursing, JHS nurses shared a view that their office should be a place, where students’ rights are respected. In Finnish health care, junior high school-aged adolescents have legal rights to determine the access to their health records (Act on the Status & Rights of Patients, 785/1992). JHS nurses did regard students’ privacy and autonomy as essential rights to be protected. JHS nurses understood that LGBTQ+ identity disclosure may take time since students needed to ensure nurse's accepting attitude to sexual and gender diversity. The nurses understanding of the importance of an accepting attitude is in line with LGBTQ+ students’ expressed needs (Laiti et al., 2021). The importance of a confidential relationship with health professionals has been identified in primary health care settings (Laiti et al., 2019). Furthermore, LGBTQ+ students have expressed, that JHS nurses could influence the school climate as LGBTQ+ advocates, and advocacy could decrease prejudice among their peers (Laiti et al., 2021). Bullying and harassment are still common experiences for LGBTQ+ students (Kosciw et al., 2020). Strategies and guidelines of LGBTQ+ advocacy in JHS nursing would be useful since JHS nurses perceived school as a conservative institution, which they can influence only minimally.

### Limitations and Strengths

We want to note two aspects related to limitations and strengths. First, we collected data by interviewing JHS nurses in focus groups with a voluntary participation. Therefore, our participants may have been school nurses, who considered the topic important, or were progressive with LGBTQ+ topics. This may limit the generalizability of findings (Morse, 2015; Patton, 2015b). Second, in qualitative research the researcher's role is significant. While qualitative research seeks to gain an understanding of people's experiences, thoughts and perceptions, it is worthy to acknowledge how researcher's interpretations can be influenced by their own identities, values, personal interests, or attitudes (Morse, 2015). In our study, the corresponding author (ML) conducted the interviews, and they acknowledged throughout the process their personal connection to LGBTQ+ community, which can influence to the interpretation. However, the corresponding author was not familiar with the work of school nurses, and they were open-minded about hearing each participant's thoughts and perceptions. Furthermore, the personal connection to LGBTQ+ community can support an in-depth understanding on sexual and gender diversity in a way, that people outside the LGBTQ+ community may be challenging to reflect.

### Implications for School Nursing Practice and Future Research

Our findings illustrated that JHS nurses’ support for LGBTQ+ students is a complex phenomenon consisting of four interconnected themes. School nurses need to be aware of, not only their professional identity and practice, but also junior high school, family acceptance, and LGBTQ+ student's position as school nursing clients. To promote LGBTQ+ inclusive school nursing and JHS work in supporting LGBTQ+ students, we recommend four areas to be developed in school nursing practice. First, it is significant to ensure school nurses’ access to evidence-based information and education on sexual and gender diversity. This topic needs to be covered both in nursing education, and in continuing education for working school nurses. Second, to enhance LGBTQ+ inclusive and safe school nursing, besides school nurses are showing accepting attitudes, the office could include safe space characteristics, such as rainbow flags and information leaflets targeted to students about LGBTQ+ topics. Third, it is essential that school nurses and other professionals are actively ensuring LGBTQ+ inclusive climate and practices in schools. Finally, as both LGBTQ+ students and JHS nurses emphasized, including family members into discussions on sexual and gender diversity is crucial to the wellbeing of LGBTQ+ students, and school nurses play an important role in the family acceptance process. This needs to happen through respecting students’ rights as school nursing clients, especially their autonomy and privacy.

Research on promoting LGBTQ+ inclusive school nursing is still lacking. Future research needs to develop educational interventions for school nurses and other nursing professionals to provide required competence on working with sexual and gender diversity topics. Furthermore, research could be done in collaboration with LGBTQ+ students through user involvement, about the development of school nursing to serve LGBTQ+ students’ needs, but also creating safe and inclusive practices.
